# Andrographolide sodium bisulphite-induced inactivation of urease: inhibitory potency, kinetics and mechanism

**DOI:** 10.1186/s12906-015-0775-4

**Published:** 2015-07-16

**Authors:** Zhi-Zhun Mo, Xiu-Fen Wang, Xie Zhang, Ji-Yan Su, Hai-Ming Chen, Yu-Hong Liu, Zhen-Biao Zhang, Jian-Hui Xie, Zi-Ren Su

**Affiliations:** Guangdong Provincial Key Laboratory of of New Chinese Medicinals Development and Research, Guangzhou University of Chinese Medicine, Guangzhou, 510006 P. R. China; Guangdong Provincial Key Laboratory of Clinical Research on Traditional Chinese Medicine Syndrome, The Second Affiliated Hospital, Guangzhou University of Chinese Medicine, Guangzhou, 510120 P. R. China; Dongguan Mathematical Engineering Academy of Chinese Medicine, Guangzhou University of Chinese Medicine, Dongguan, 523000 P. R. China

**Keywords:** Andrographolide sodium bisulphite, Urease, Inhibition, Slow-binding, Sulfhydryl group, Molecular docking

## Abstract

**Background:**

The inhibitory effect of andrographolide sodium bisulphite (ASB) on jack bean urease (JBU) and *Helicobacter pylori* urease (HPU) was performed to elucidate the inhibitory potency, kinetics and mechanism of inhibition in 20 mM phosphate buffer, pH 7.0, 2 mM EDTA, 25 °C.

**Methods:**

The ammonia formations, indicator of urease activity, were examined using modified spectrophotometric Berthelot (phenol-hypochlorite) method. The inhibitory effect of ASB was characterized with IC_50_ values. Lineweaver-Burk and Dixon plots for JBU inhibition of ASB was constructed from the kinetic data. SH-blocking reagents and competitive active site Ni^2+^ binding inhibitors were employed for mechanism study. Molecular docking technique was used to provide some information on binding conformations as well as confirm the inhibition mode.

**Results:**

The IC_50_ of ASB against JBU and HPU was 3.28 ± 0.13 mM and 3.17 ± 0.34 mM, respectively. The inhibition proved to be competitive and concentration- dependent in a slow-binding progress. The rapid formation of initial ASB-JBU complex with an inhibition constant of *K*_*i*_ = 2.86 × 10^−3^ mM was followed by a slow isomerization into the final complex with an overall inhibition constant of *K*_i_* = 1.33 × 10^−4^ mM. The protective experiment proved that the urease active site is involved in the binding of ASB. Thiol reagents (L-cysteine and dithiothreithol) strongly protect the enzyme from the loss of enzymatic activity, while boric acid and fluoride show weaker protection, indicating that the active-site sulfhydryl group of JBU was potentially involved in the blocking process. Moreover, inhibition of ASB proved to be reversible since ASB-inactivated JBU could be reactivated by dithiothreitol application. Molecular docking assay suggested that ASB made contacts with the important sulfhydryl group Cys-592 residue and restricted the mobility of the active-site flap.

**Conclusions:**

ASB was a competitive inhibitor targeting thiol groups of urease in a slow-binding manner both reversibly and concentration-dependently, serving as a promising urease inhibitor for the treatment of urease-related diseases.

## Background

Urease (urea amidohydrolase, EC 3.5.1.5) which catalyzes the hydrolysis of urea to produce ammonia and carbon dioxide has been found in plants, algae, fungi, bacteria and soil [1]. It is a thiol-rich and nickel-dependent metalloenzyme that can catalyze the hydrolysis of urea, thereby producing ammonia and carbamate [2]. In addition to the archetypical nickel-containing urease, *Helicobacter mustelae*, a gastric pathogen of ferrets, was recently found to synthesize a distinct iron-dependent oxygen-labile urease with dinuclear Fe and less activity [3]. Nevertheless, the nickel ions (Ni^**2+**^) and the sulfhydryl group, especially the multiple cysteinyl residues in the active site of the enzyme, are essential for the catalytic activity of all ureases. As the reaction results in the increase of pH values, urease is responsible for negative effects of urease activity in human health and agriculture.

*Helicobacter pylori* (*H. pylori*) is a well-established etiologic agent of gastritis, gastric and duodenal ulceration, even gastric carcinoma [4]. *H. pylori* urease (HPU), a highly active urease produced by *H. pylori*, is a virulence factor in infections of gastrointestinal tracts. HPU could initiate the hydrolysis of urea generating ammonia, which allows the bacterium to survive and colonize the low pH environment of the gastric mucosa [5]. Therefore, strategies based on urease inhibition are now considered as the first line of treatment for infections caused by urease-producing microorganisms.

In past decades, varieties of urease inhibitors have been investigated including phosphoramidates [2], hydroxamic acids [6], boric and boronic acids [7], heavy metal ions [8], quinones [9] and imidazoles [10]. However, most of these compounds are too toxic or unstable to be therapeutic agents. Thus, current researches are focused on finding novel urease inhibitors with promising levels of activity from natural plant.

**Fig. 1:**
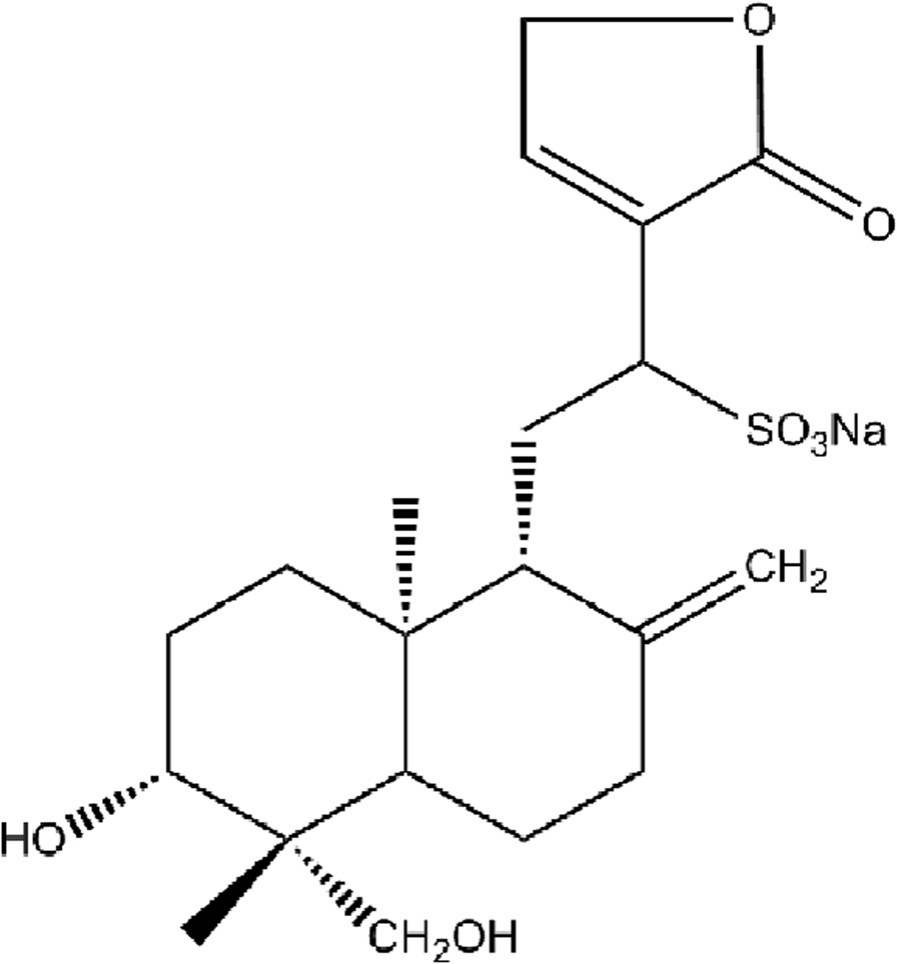
Chemical structure of ASB

Andrographolide (C_20_H_30_O_5_), the major diterpenoid lactone and the primary effective constituent of *Andrographis paniculata* (a widely used Chinese medicinal herb known as ‘Chuan-Xin-Lian’ in Chinese), has multiple pharmacological properties, including antimicrobial [11], anti-inflammation [12, 13], anti–cancer [14] and immunity enhancement [15–17]. Andrographis paniculata was reported to posses anti-*H. pylori* activity [18] and effectively relieve *H. pylori* associated gastritis in clinical practice [19, 20]. In addition, a variety of andrographolide derivatives proved to exert inhibitory effects on enzymes [21–23]. Therefore, andrographolide is expected to exert inhibitory properties against urease, counteracting the undesirable effects brought about by activated urease. Jack bean urease (JBU) is the best-characterized [24–26] and widely-employed instrumental enzyme in urease inhibition research [27, 28]. Additionally, it has been found that the inhibition mechanism of action and kinetics of inhibition for bacteria urease and JBU are similar [29]. In the present investigation, the inhibitory effect against JBU of andrographolide sodium bisulphite (ASB, C_20_H_29_O_7_S · Na, shown in Fig. 1), a water-soluble sulfonate of andrographolide, was performed to elucidate the kinetics and mechanism of inhibition.

## Methods

### Materials and reagents

Andrographolide sodium bisulfite (C_20_H_29_O_7_S · Na, CAS number 71202-97-6), urea (molecular biology reagent), D,L-dithiothreitol (DTT) , L-cysteine (L-cys), boric acid and sodium fluoride (NaF) were purchased from Sigma Aldrich. JBU (from jack bean, *Canavalia ensiformis*, type III, nominal activity 40.3 units/mg, solid) was also from Sigma Aldrich, of which one unit of urease activity is defined as the amount of enzyme needed to liberate 1.0 μmol of NH_3_ from urea per min at pH 7.0 at 25 °C. Brucella broth was purchased from Becton–Dickinson. (Cockeysville, MD). Other chemicals were obtained from Guangzhou Chemical Reagent Factory (China). All reagents were of analytical grade. Phosphate buffer (PBS, 20 mM, pH 7.0) was prepared by adjusting pH of phosphoric acid with NaOH. 2 mM EDTA was added to all enzyme-containing solutions.

### Preparation of *H. pylori* urease

*Helicobacter pylori* (ATCC 43504; American Type Culture Collection, Manassas, VA) was grown in brucella broth supplemented with 10 % heat-inactivated horse serum for 24 h at 37 °C under microaerobic conditions (5 % O_2_, 10 % CO_2_, and 85 % N_2_) [30, 31]. For urease inhibition assays, 50 ml broth cultures (2.0 × 10^8^ CFU/mL) were centrifuged (5000 g, 4 °C) to collect the bacteria, and after washing twice with phosphate-buffered saline (pH 7.4), the *H. pylori* precipitation was stored at -80 °C. *H. pylori* was returned to room temperature, and after addition of 3 mL of distilled water and protease inhibitors, sonication was performed for 60 s. Following centrifugation (15,000 g, 4 °C), the supernatant was desalted through Sephadex G-25 column (PD-10 columns, Amersham–Pharmacia Biotech, Uppsala, Sweden). The resultant crude urease solution was added to an equal volume of glycerol and stored at 4 °C until use in the experiment.

### Standard urease activity assay

The standard urease assay mixture contained 50 mM urea in 20 mM phosphate buffer (pH 7.0) containing 2 mM EDTA. After addition of the enzyme-containing solution of 0.25 mg/mL JBU, the assay ran for 20 min, and the enzyme activity was determined by measuring the concentration of the ammonia released in the reaction mixture. For ammonia measurement, aliquots were withdrawn from the reaction mixtures, and the ammonia was determined at 595 nm spectrophotometrically according to the modified Berthelot (phenol-hypochlorite) method [32] at ambient temperature.

### Inactivation of JBU by ASB

Urease solutions mixed with serial concentrations of ASB ( 0–6 mM) were incubated at 37 °C for 20 min, which contained 0.25 mg/mL JBU, 20 mM phosphate buffer (pH 7.0), and 2 mM EDTA. The initial time of incubation was defined as the moment once the enzyme and inhibitor were mixed. After appropriate period of time, aliquots from the incubation mixture were transferred into the standard assay mixtures for urease residual activity determination. The activity of uninhibited urease was defined as the control activity of 100 %.

### Determination of *K*_*M*_ and *v*_*max*_

The Michaelis constant *K*_*M*_ and the maximum velocity *v*_*max*_ in the absence of the inhibitor were determined by measuring the initial reaction velocities at different urea concentrations ranging from 0.4 to 10 mM. The values were obtained by applying nonlinear regression to the Michaelis-Menten equation.

### Reaction progress curves monitoring

The reaction progress was studied in the absence or presence of ASB using the following two procedures.

- Unpreincubated System. The progress curves were determined by the reactions directly initiated by the addition of the JBU into the reaction mixtures containing different concentrations of ASB (2, 4 and 6 mM).
- Preincubated System. The JBU was preincubated with ASB for 20 min first, and the reaction was then initiated by addition of urea solution into the reaction preincubation mixtures containing different concentrations of ASB (2, 4 and 6 m.M).

JBU activities in both procedures were determined as described in standard urease activity assay. A curve-fitting computer program was employed to fit the experimental points to the integrated equation describing slow-binding inhibition progress curves [7]:

1 $$ p(t)={v}_st+\frac{\left({v}_0-{v}_s\right)\left(1-{e}^{-{k}_{ap{p}^t}}\right)}{k_{app}} $$

where *p* is the amount of product accumulated at time *t* after initiation of the reaction. *v*_*0*_ and *v*_*s*_ are the reaction initial and steady-state velocities, respectively, and *k*_*app*_ denotes the apparent first-order velocity constant for interconversion between *v*_*0*_ and *v*_*s*_.

### JBU protection against ASB inactivation

This part of the study was carried out as follows. JBU was first preincubated with different protectors for 20 min. Then, samples of the protected JBU were incubated with 4 mM ASB for additional 20 min. The urease activity was assayed upon incubation of the mixture. For protection by boric acid and NaF, JBU was preincubated with 4 mM boric acid and 4 mM NaF, respectively. For protection by thiols, the applied thiol-containing compounds (L-cys and DTT) were of a series of concentrations.

### ASB -thiol-urease interaction test

The incubation mixtures contained JBU solution, ASB, and mono thiols (L-cys) or dithiol (DTT). The components of the incubation mixture were mixed according to the following three procedures:

- JBU was added to the mixture after a 20 min contact of ASB with the thiol.
- ASB was added to the mixture after a 20 min contact of JBU with thiol.
- Thiol was added to the mixture after a 20 min contact of JBU with ASB.

The complete mixture was mixed thoroughly and incubated for additional 5, 10, 20, and 40 min. Then, JBU activity assay was determined as described in the inactivation of JBU by ASB.

### Reactivation of ASB -inactivated JBU

The reactivation of inactivated JBU was studied in two ways: by using DTT, L-cys and by multidilution in the reaction mixture containing urea.

- After a 20 min preincubation of JBU with ASB (4 mM), the mixture was further incubated with DTT or L-cys for 120 min. The activity of JBU was determined before and after the addition of DTT.
- ASB (4 mM) was preincubated for 10 and 20 min, respectively, with the enzyme to establish the equilibrium: $E$ + $I$ ⇔ $EI$ ⇔ $EI$ ∗, and then the preincubation mixture was diluted 50 folds into the reaction mixture. After appropriate period of time, aliquots were withdrawn, and the amount of ammonia was determined.

### Molecular docking

The automated docking studies were carried out using Auto-Dock version 4.0 as implemented through the graphical user interface AutoDock Tools (ADT 1.5.2). The three-dimensional crystal structure of JBU (PDB code: 3LA4) was obtained from the RCSB Protein Data Bank,whose resolution was 2.05 Å . The required actions were to remove water molecules from the protein, add all hydrogen atoms, calculate Gasteiger charges, and merge nonpolar hydrogen atoms to carbon atoms. The standard 3D structure (PDB format) of ASB was obtained with chem3D Ultra 8.0 software. The PDB files were further transferred to PDBQT files with AutoDock Tools. The three-dimensional results were created by the PyMol molecular graphics system [33]. The cubic grid box of 60 Å size (*x, y, z* ) with a spacing of 0.5 Å and grid maps were built. The center of the grid was set to the average coordinates of the two Ni^2+^ ions. The Lamarckian genetic algorithm (LGA) was selected as the search algorithm. The Lamarckian job consisted of 100 runs. Default settings were used with an initial population of 150 randomly placed individuals, a maximum number of 2.5 × 10^6^ energy evaluations, and a maximum number of 2.7 × 10^4^ generations. A mutation rate of 0.02 and a crossover rate of 0.8 were chosen. Van der Waals and hydrogen bonding were included in the calculated non-bonded energy. Results differing by less than 0.5 Å in positional root-mean-square deviation (RMSD) were clustered together, and the results of the most favorable free energy of binding were chosen as the resultant complex structures.

## Results and discussion

### Urease inhibition assays

**Fig. 2:**
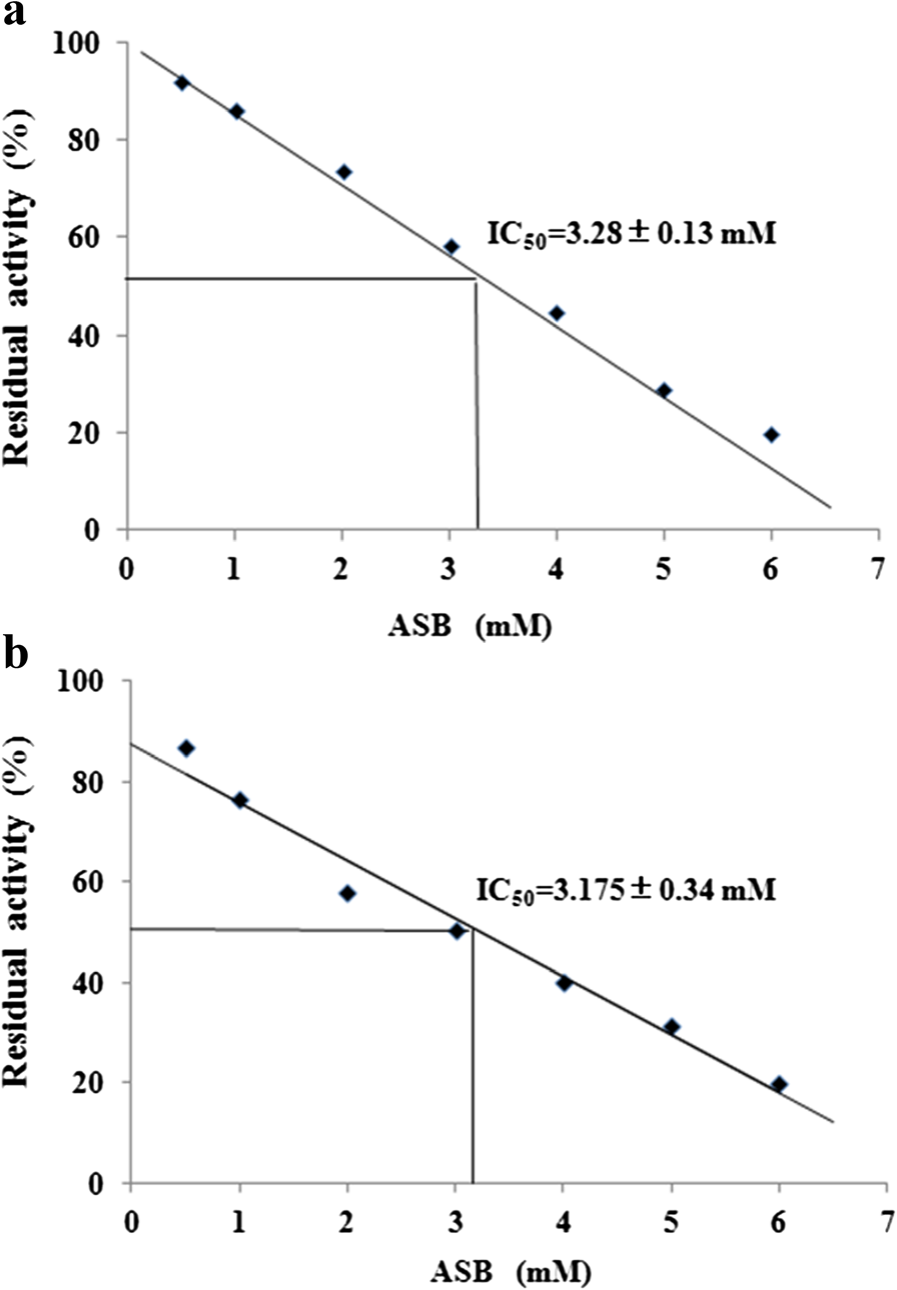
Dependence of residual activity versus concentration of ASB. **a** residual activity of JBU inhibited by ASB; **b** residual activity of HPU inhibited by ASB

The system of jack bean urease-ASB-urea, can be treated as a model system in the studies on the utility of ASB in the therapy of diseases caused by *H. pylori* and other bacteria producing urease. Data from Fig. 2 depicted urease residual activity as a function of ASB concentration. The IC_50_ indicated the ASB concentration that could descend the activity of urease to 50 %. The obtained IC_50_ value of ASB against HPU and JBU was 3.17 ± 0.34 mM (*R*^2^ = 0.976) and 3.28 ± 0.13 mM (*R*^2^ = 0.997), respectively. The linear function for this relation was a good-enough approximation. Based on the above mentioned, a conclusion could be drawn that ASB performed similar effectiveness on the inhibition of these two ureases.

### Kinetics of JBU inactivation by ASB

**Fig. 3:**
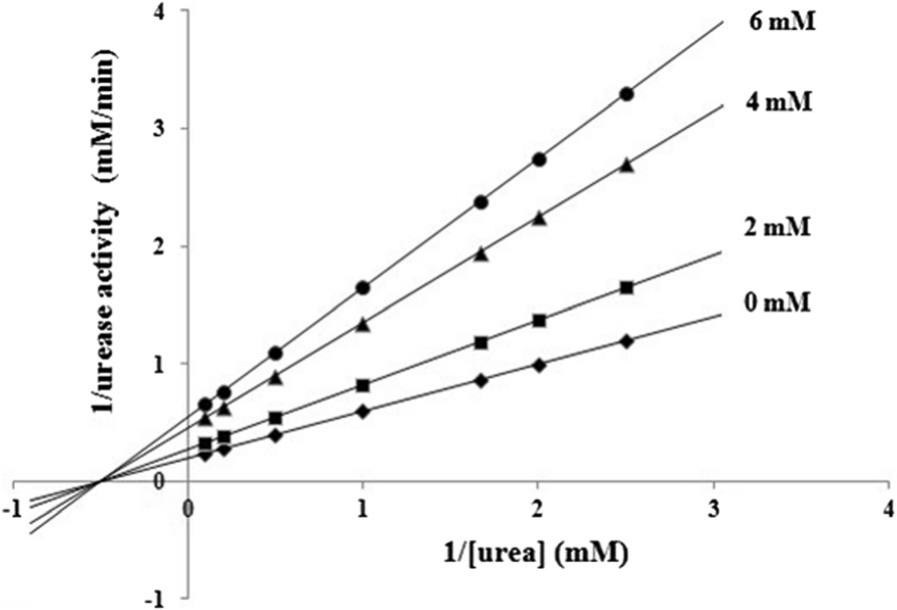
Lineweaver-Burk plot of the reciprocal of urease activity versus reciprocal of substrate concentration in the absence and presence of 2 mM, 4 mM, and 6 mM of ASB

**Fig. 4:**
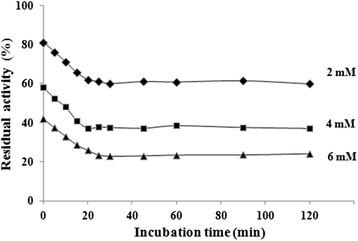
Dependence of residual activity versus preincubation time with ASB. Concentration of ASB (mM) is numerically given

Enzyme kinetics was determined in the absence and presence of various concentrations of ASB. *K*_*M*_ and *v*_*max*_ of ureolytic reaction by applying nonlinear regression to the Michaelis-Menten equation were 2.001 ± 0.08 mM and 0.125 ± 0.07 mM/min, respectively. As the Lineweaver-Burk plots for ASB shown in Fig. 3, *K*_*M*_ value did not significantly change in the presence of ASB, while the *V*_*max*_ value decreased as the ASB concentration increased, indicating that the effect of ASB on JBU might be a noncompetitive mechanism of inhibition. Additionally, our data indicated a slow-binding inhibition relationship of enzyme activity *versus* preincubation time [9, 34], which indicated the total urease activity in the free form and in the form of being bound in the urease inhibitor complexes *EI* and EI*. It was clear in Fig. 4 that increasing the preincubation time resulted in a decrease of urease activity. The activity descended rapidly at the beginning until the equilibrium between urease (E), inhibitor (I), and urease-inhibitor complexes (*EI*) and (EI*) (*E + I* ⇔ *EI* ⇔ EI* ) was achieved, which was characterized by the constant urease activity, since the slow-binding effect would not be revealed unless the enzyme interacted with the inhibitor for sufficient time. Otherwise, it would lead to a misinterpretation as a noncompetitive type if determined by the initial reaction rates method. Hence, the progress curves analysis was employed to confirm the slow-binding model of JBU inactivation by ASB.

### Progress curves analysis

**Fig. 5:**
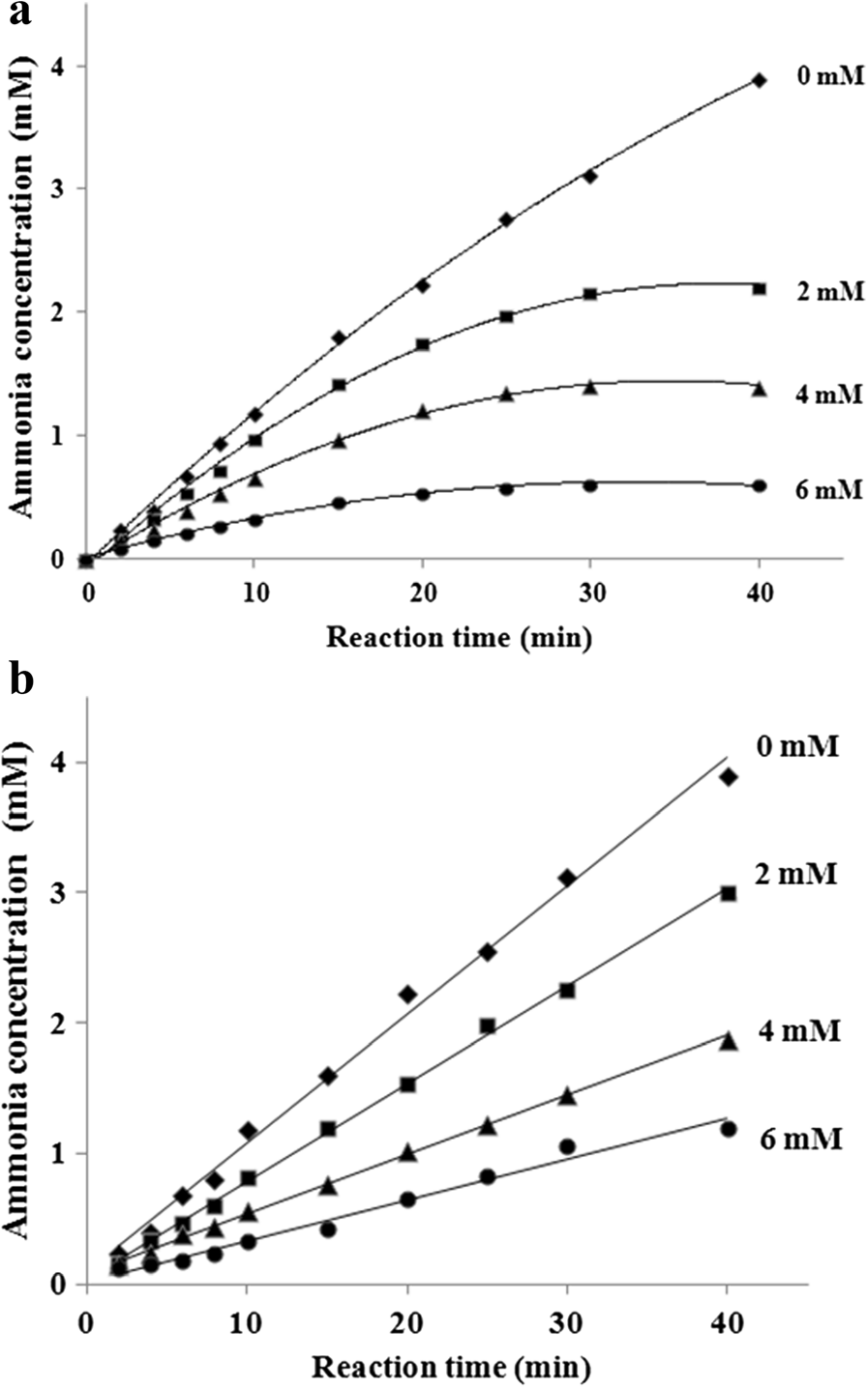
**a** Reaction progress curves of urease-catalyzed hydrolysis of urea in the presence of ASB; **b** Steady-state analysis: concentration of ammonia versus time. ASB concentration (mM) is numerically given

The progress curves for urea hydrolysis under ASB-inhibited JBU catalyzation were shown in Fig. 5. The reaction progress curves for the unpreincubated system were concave downward (Fig. 5(a)), indicating that the velocity of urea hydrolysis decreased from an initial velocity (*v*_*0*_) to a much slower steady-state velocity (*v*_*s*_) according to the apparent first-order velocity constant *k*_*app*_. Such a behavior is characteristic of slow-binding inhibition elaborated by the theory of Morrison and Walsh. The obtained results also showed that the *v*_*0*_ and *v*_*s*_ were inhibitor concentration-dependent. In terms of the preincubation system (steady-state analysis, Fig. 5(b)), the linear curves proved that the reaction achieved the steady-state velocity (*v*_*s*_), being different from each studied inhibitor concentration.

The obtained relationship of the reaction velocities (*v*_*0*_; *v*_*s*_) *versus* the inhibitor concentration is characteristic of a two-step enzyme inhibitor interaction, mechanism B described as follows,

2 $$ \begin{array}{l}E+S\underset{k_2}{\overset{k_1}{\leftrightarrow }}\kern0.2em ES\overset{k^7}{\to }E+P\\ {}E+I\underset{k_4}{\overset{k_3}{\leftrightarrow }}\kern0.5em \underset{\mathrm{slow}}{\underbrace{EI\underset{k_6}{\overset{k_5}{\leftrightarrow }}EI\;}}*\end{array} $$

where *E* is enzyme, *S* is substrate, *P* is product, *I* is inhibitor, and *EI* and EI* are enzyme-inhibitor complexes, respectively. *k*_*1*_*-k*_*7*_ are velocity constants. Linear dependencies of *1/v*_*0*_ and *1/v*_*s*_ on the inhibitor concentration are used to evaluate the inhibition constants, *K*_*i*_ and *K*_i_*, as follows:

3 $$ \begin{array}{l}\frac{1}{vo}=\frac{KM}{v \max SoKi}I+\frac{1}{v \max}\left(1+\frac{KM}{So}\right)\\ {}\frac{1}{vs}=\frac{KM}{v \max SoKi*}I+\frac{1}{v \max}\left(1+\frac{KM}{So}\right)\end{array} $$

where *K*_*M*_ is the Michaelis constant and *v*_*max*_ is the maximum velocity given by the Michaelis-Menten equation for the uninhibited reaction; *S*_*0*_ denotes the initial concentration of urea; *K*_*i*_ and *K*_i_* are the inhibition constants defined as: *K*_*i =*_ [*E*] [*I*] / [*EI*] and *K*_i_* = [*E*] [*I*] / ( [*EI*] + [EI*] ) respectively.

By calculating from reciprocal dependence of *v*_*0*_ and *v*_*s*_ on the inhibitor concentration according to formula [3], it was found that the initial ASB-JBU complex formed rapidly with an inhibition constant of *K*_*i*_ = (2.86 ± 0.09) × 10^−3^ mM, followed by a slow isomerization into the final ASB-urease complex with the overall inhibition constant of *K*_i_* = (1.33 ± 0.11) × 10^−4^ mM. The rate constant of the ASB-JBU isomerization indicated that forward process was rapid in contrast with slow reverse reactions. The overall inhibition constant obtained by the steady-state analysis was (1.18 ± 0.13) × 10^−3^ mM. Furthermore, the shape of the curves in that case corresponded to the competitive slow-binding type of inhibition, as represented by formula [1]. In details, the reaction was inhibited slightly in the initial period, characterized by high reaction rates *v*_*0*_. Then, in the later period, the inhibition became stronger, characterized by lower reaction rates *v*_*s*_. This indicated a competitive inhibition in both initial and steady-state stages of the inhibition reaction.

Taken together, the progress curves analysis and preincubation studies proved that the ASB inhibition on urease was indeed in a slow-binding and competitive manner.

### Urease protection against ASB inactivation

**Fig. 6:**
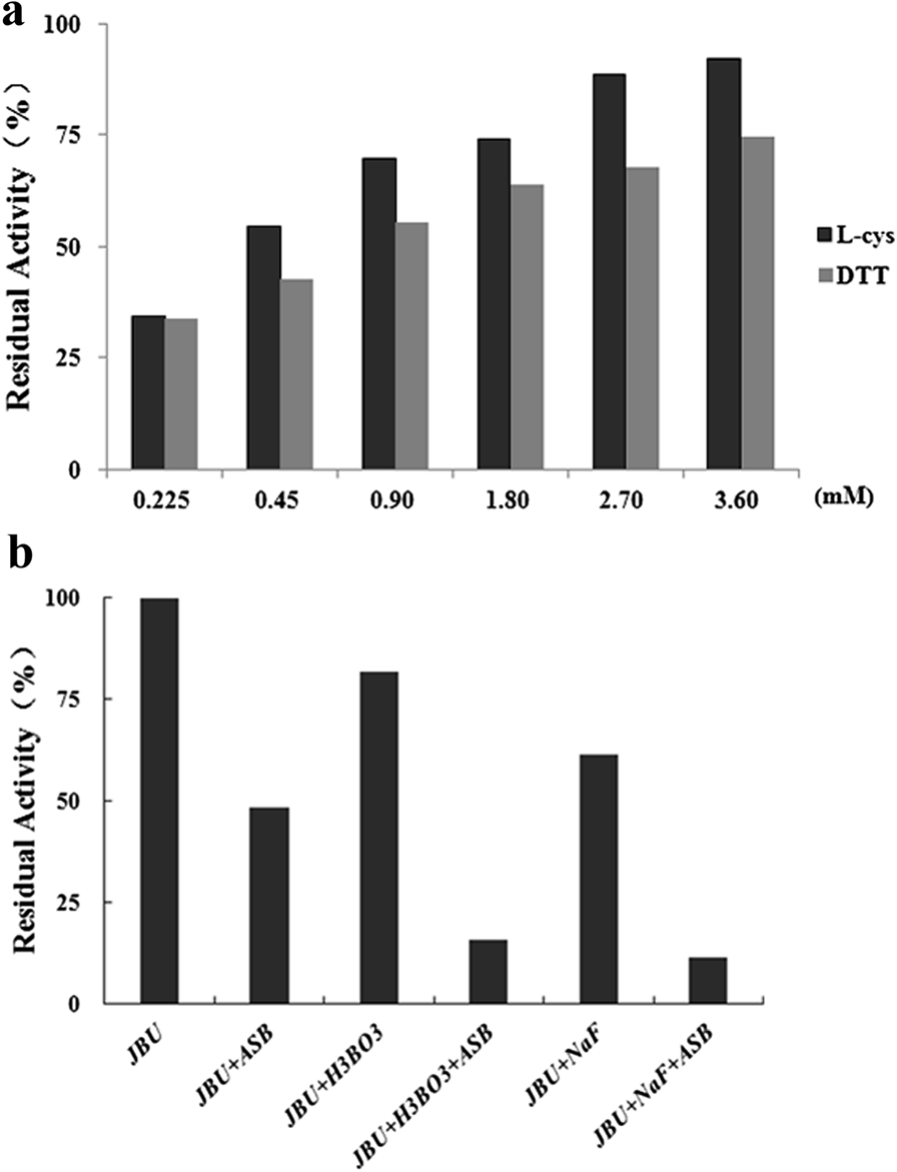
**a** L-cys and DTT protection of urease against ASB. Concentration of L-cys and DTT (mM) is numerically given; **b** Protection of urease against ASB inactivation by boric acid and sodium fluoride

Up to now, it has been found that there were two well-defined urease protectors. One is the inorganic compounds (NaF and boric acid) reacting with active-site nickel ions, and the other is thiol-containing compounds such as DTT and L-cys, which interact with sulfhydryl groups of urease [35, 36]. When equilibrated with the enzyme, the protectors by occupying the active site restrict the accessibility of inhibitions to the active-site functional groups. Hence, in the present investigation, both protectors were employed to investigate the possible inhibition target of ASB. According to Fig. 6(a), the urease protection effect against inactivation by ASB was enhanced as the concentration of thiol reagents increased. After the inactivation by 4 mM ASB, L-cys and DTT could restore the urease activity in a concentration-dependent manner. This indicated that the thiol groups were involved in the inactivation of the enzyme and that there was a better affinity of ASB towards L-cys and DTT than the thiol group in urease. Meanwhile, the protective potency of L-cys was found stronger than that of DTT. This might be due to the pH of the reaction system (pH = 6.9-7.0), in which DTT failed to exert the desired protective effect [37]. By contrast, protections with sodium fluoride (a competitive slow-binding urease inhibitor) [35] and boric acid (a classical competitive urease inhibitor) [35, 36] were relatively insignificant. As Fig. 6(b) demonstrated, when urease was inactivated by ASB in the presence of sodium fluoride and boric acid, the enzymatic activity decreased to 11.42 % and 15.61 %, respectively, even lower than that in the presence of ASB alone, suggesting a probable synergic relationship between ASB and sodium fluoride or boric acid. Obviously, better prevention by thiols than by inorganic compounds against ASB inactivation indicated that the active-site sulfhydryl group might be a residue responsible for urease inhibition.

### ASB-thiol-urease interaction test

**Fig. 7:**
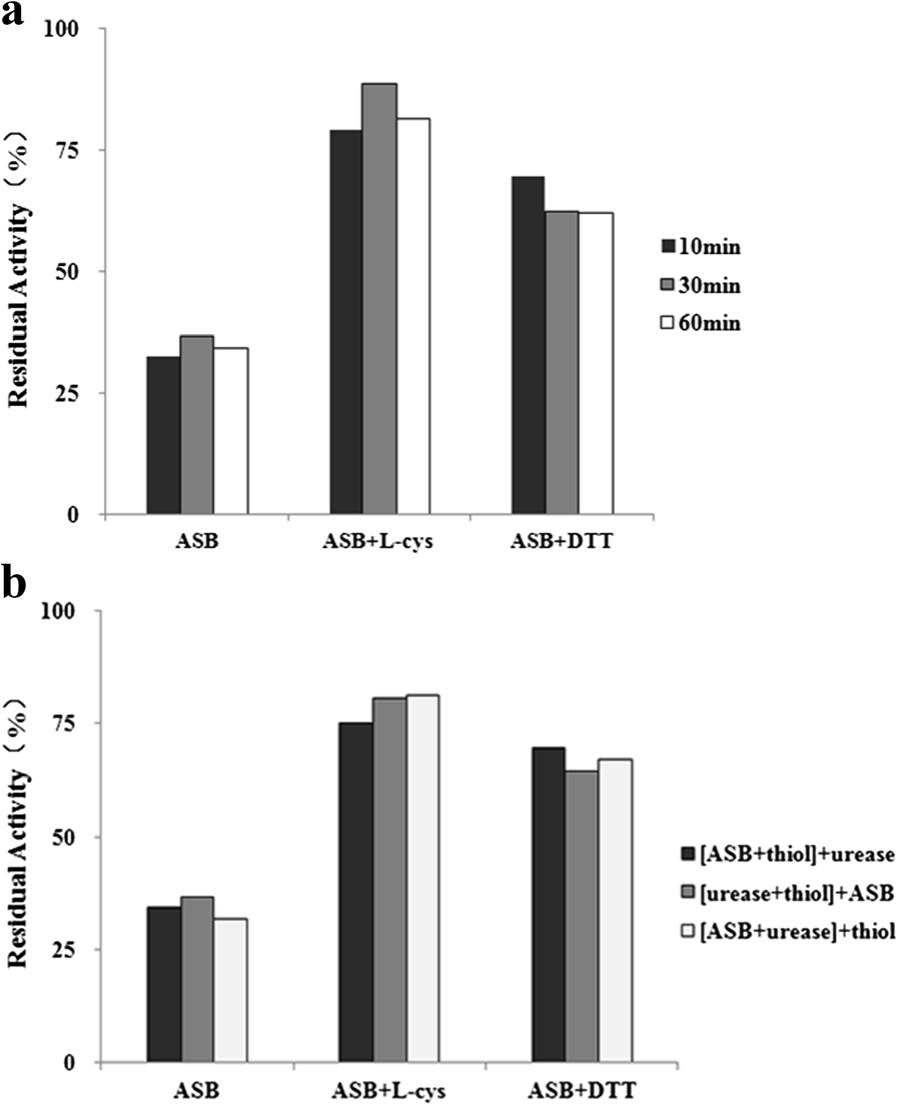
**a** Thiol influence on urease inactivation by ASB relative to the control activity. The percent of the enzyme activity in the presence of ASB without the thiol is given as comparison. Concentration of the thiol: L-cys, DTT, and ASB were 4 mM. Enzyme activity was determined after 10, 30, 60 min of incubation; **b** Influence of thiol order of components preincubation on urease inactivation by ASB. The initial 20 min preincubation mixture contained components given in brackets. The preincubation was continued the further 20 min after addition of the last component (component given outside for brackets). The final preincubation mixtures contained 0.25 mg/mL urease, 20 mM phosphate buffer, pH 7.0, 2 mM EDTA, 4 mM ASB, and DTT or L-cys. Enzyme activity was determined after 40 min of preincubation time. The percent of the enzyme activity in the presence of ASB without the thiol is given for comparison

The role of thiols in ASB inactivation was studied by comparing urease activities in thiol-free system at three time points of incubation. It was found that monothiol (L-cys) and dithiol (DTT) could alleviate the inactivation by ASB, and urease regained activity in spite of ASB presenting in the incubation mixture. When the thiol-containing compounds provided thiol groups, concentration was equal or higher than that of ASB. However, as Fig. 7(a and b) shown, incubation time had no significant effect on the ASB-thiol-urease interaction. And the protection potency remained consistent regardless of the addition order of urease, inhibitors, and protectors.

The presence of the thiol-protector in the incubation system allowed ASB to react with thiols from the JBU and those in the “free” thiol-protector. The thiols presenting in the protein were much less reactive than those presenting externally in the form of L-cys or DTT. The decreases of urease activity in the thiol-free system and system with the thiols were compared, suggesting that the general losses of urease activity in both systems remained, but it was slowed down in the presence of thiols, especially in the presence of L-cys. These data suggested that ASB-thiol interaction was strategic for the inactivation rate decrease.

### Reactivation of ASB-inactivated urease

**Fig. 8:**
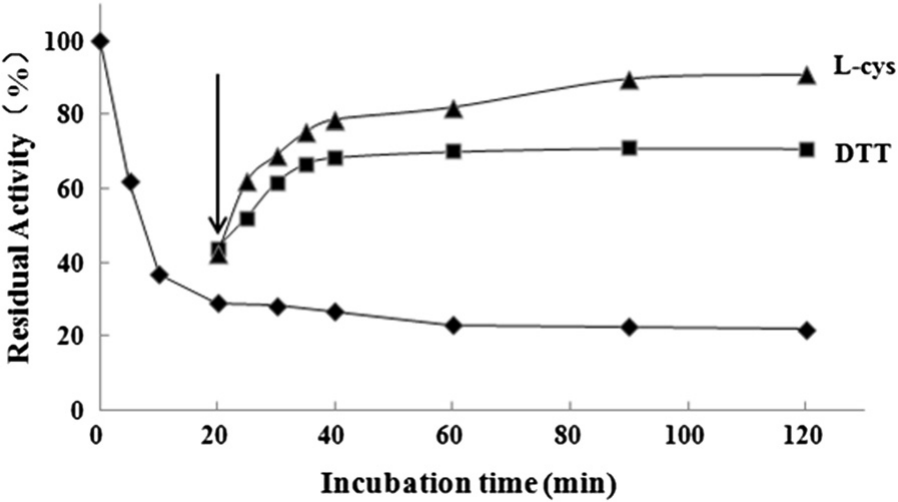
Reactivation of ASB-inactivated urease by L-cys or DTT. Activity of urease inactivated by ASB (blue line) and after adding L-cys (yellow) or DTT (purple). Urease was inactivated by 4 mM ASB, and 4 mM L-cys or DTT was added into the reaction system 20 min later (as indicated by the vertical arrow)

To investigate whether the inactivation of JBU by ASB was reversible, the reactivation of ASB-inactivated JBU was carried out in two ways. In the first way, by addition of L-cys or DTT after the 20 min incubation of JBU with ASB, urease activity recovered in a time-dependent manner: after 1.5 h, JBU had restored 90.77 % and 70.98 % of its initial activity, respectively (Fig. 8). After reactivated by L-cys or DTT, retreatment of ASB could not inhibit the urease activity again. This evidence indicated that the urease-ASB complex was less resistant for chemical approach.

By contrast, by multidilution, urease remained in constant activity as the concentration of ammonia increased, which indicated that an insignificant amount of the active enzyme separated from urease-ASB complex after dilution due to no further release of active urease (data not shown). Taken together, there would be a supposed reversibility between urease and ASB, in which the chemical approach but not multidilution could recover the enzyme activity that had been inactivated by ASB.

### Molecular docking

**Fig. 9:**
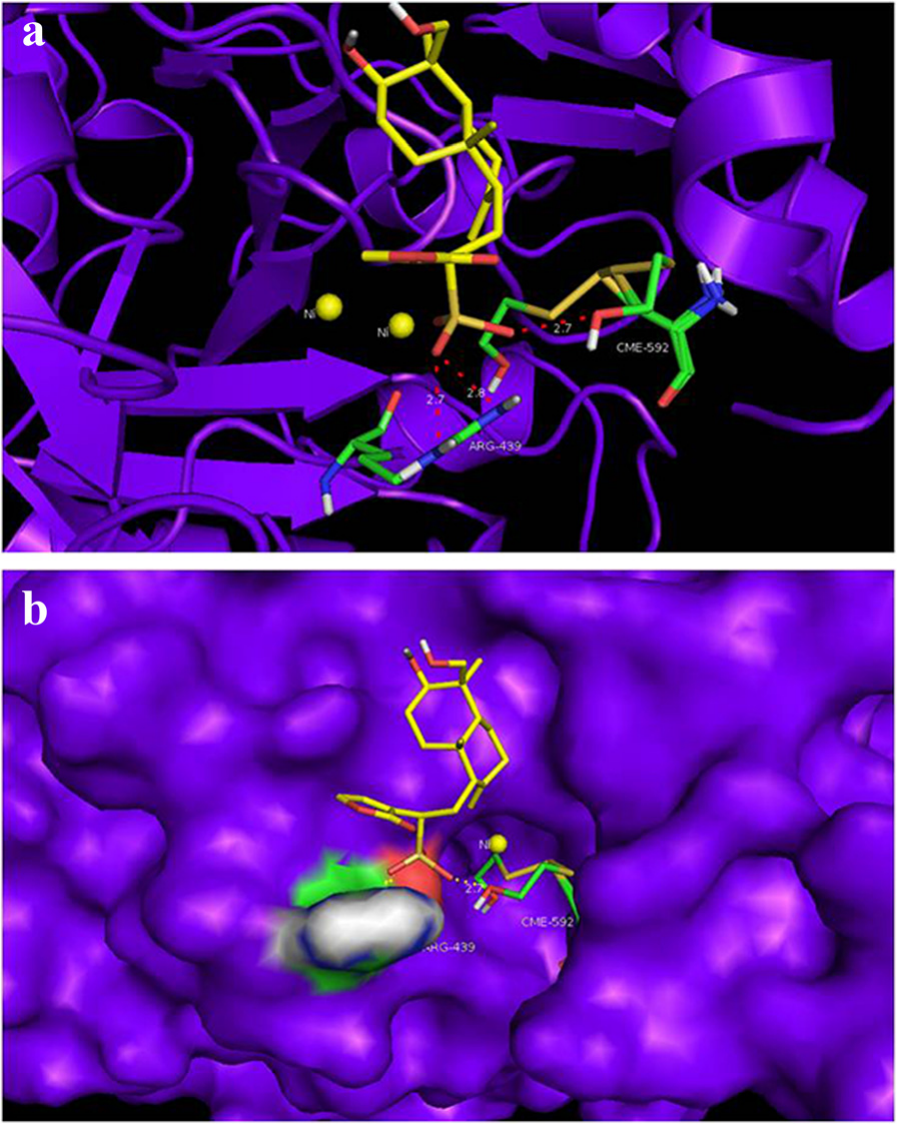
**a** Molecular docking simulations obtained at the lowest energy conformation, highlighting potential hydrogen contacts of ASB. For clarity, only interacting residues are labeled. Hydrogen bonding interactions are shown by dashes; **b** Surface representation of the active-site flap of JBU with ASB shown at the entrance of the binding pocket. (colored by atom: carbon is yellow; oxygen is red; hydrogen is white; sulfur is brown; nitrogen was blue)

In order to elucidate the inhibition mechanism revealed by the kinetics study, molecular docking of ASB into the crystal structure of JBU (3LA4 in the Protein Data bank) was performed by the AutoDock program, and the best possible binding modes were shown in Fig. 9.

In the best possible binding mode, ASB tightly anchored the helix-turn-helix motif over the active-site cavity through O − H∙ ∙ ∙S, N − H∙ ∙ ∙O, and O − H∙ ∙ ∙O hydrogen bonding interactions. This mode made ASB engage a cleft beside the active-site cavity, using 3 typical hydrogen bonds to anchor the flap tightly with the backbone of the enzyme, thereby preventing the flap from backing to the close position. The 12- SO_3_H of ASB, as the hydrogen bond donor ,was found between the OH and the backbone S atom of CME 592 (H∙ ∙ ∙S distance = 2.7 Å), which was located on the mobile flap closing the active site of the enzyme. In addition, the 12- SO_3_H of ASB was bound via two hydrogen bonds to NH_2_ of ARG 439 with O∙∙∙H distance of 2.7 Å and 2.8 Å, respectively. The Cys-592 (marked as CME592) is a key residue located at the mobile flap covering the active site, one per each of the six sites in the hexameric molecule [38]. Besides being directly involved in the architecture of the active site, the residue has a vital role in positioning other key residues in the active site appropriately for the catalysis [28, 39]. The flexible flap goes through an open-closed-open procedure, effectively activating the inert urea leading to activated enzyme during the normal urea catalyzed by urease [40]. And modification of Cys-592 resulted in restriction of the mobility of the flap, subsequently perturbed the reaction, and reduced enzymatic activity. In addition, ARG-439, the other residue of the flap at the entrance of the binding pocket, participate in the substrate binding, stabilize the catalytic transition state, and accelerate the reaction mainly through hydrogen bonding. It was reported that some urease inhibitors depressed JBU activities by interacting with the sulfhydryl group of residues, especially the Cys-592 [41].

As the results described, ASB possibly made hydrogen bonding interactions with the side chains of the above-mentioned residues, especially the active-site flap Cys-592, hence preserving the flap in an open conformation and resulting in an inactivation. The observation was soundly supportive of the earlier conclusion drawn from the urease protection experiments performed with the active-site binding inhibitors, which supported the observation that inhibition by ASB involved the participation of sulfhydryl group of the active-site cysteine. Taking into account the peculiarities of the active-site flap cysteine in the urease catalysis and sulfhydryl group in urease activity, it could be inferred that ASB made contacts with the side chains of cysteine residues, especially sulfhydryl group, which was reflected in their enhanced affinity to the Cys-592. As a result, the mobility of flap was restricted, and the enzymatic activity significantly declined finally.

## Conclusions

Based on the research on the mechanism and kinetics of urease inhibition by ASB in the present study, it could be concluded that ASB was a competitive inhibitor targeting thiol groups in the active site of urease in a slow-binding manner, both concentration dependent and reversible. Hence, ASB deserves to be further exploited as a promising urease inhibitor for treatments of urease-related diseases.

## Abbreviations

ASB: Andrographolide sodium bisulphite
JBU: Jack bean urease
HPU: *Helicobacter pylori* urease
DTT: D,L-dithiothreitol
L-cys: L-cysteine
NaF: Sodium fluoride
EDTA: Ethylene diamine tetraacetic acid
PBS: Phosphate buffer
LGA: The Lamarckian genetic algorithm
RMSD: Root-mean-square deviation
